# To Be or Not to Be? Are Reactive Oxygen Species, Antioxidants, and Stress Signalling Universal Determinants of Life or Death?

**DOI:** 10.3390/cells11244105

**Published:** 2022-12-17

**Authors:** Magdalena Szechyńska-Hebda, Roshanak Zarrin Ghalami, Muhammad Kamran, Frank Van Breusegem, Stanisław Karpiński

**Affiliations:** 1 Department of Plant Genetics, Breeding and Biotechnology, Institute of Biology, Warsaw University of Life Sciences, Nowoursynowska 159, 02-776 Warsaw, Poland; 2 W. Szafer Institute of Botany of the Polish Academy of Sciences, Lubicz 46, 31-512 Kraków, Poland; 3 UGent Department of Plant Biotechnology and Bioinformatics, VIB-UGent Center for Plant Systems Biology Ghent University, Technologiepark-Zwijnaarde 71, 9052 Ghent, Belgium

**Keywords:** cell death, cellular light memory, hormonal and electrical signalling, network acquired acclimation, ROS signalling, systemic acquired acclimation, systemic acquired resistance

## Abstract

In the environmental and organism context, oxidative stress is complex and unavoidable. Organisms simultaneously cope with a various combination of stress factors in natural conditions. For example, excess light stress is accompanied by UV stress, heat shock stress, and/or water stress. Reactive oxygen species (ROS) and antioxidant molecules, coordinated by electrical signalling (ES), are an integral part of the stress signalling network in cells and organisms. They together regulate gene expression to redirect energy to growth, acclimation, or defence, and thereby, determine cellular stress memory and stress crosstalk. In plants, both abiotic and biotic stress increase energy quenching, photorespiration, stomatal closure, and leaf temperature, while toning down photosynthesis and transpiration. Locally applied stress induces ES, ROS, retrograde signalling, cell death, and cellular light memory, then acclimation and defence responses in the local organs, whole plant, or even plant community (systemic acquired acclimation, systemic acquired resistance, network acquired acclimation). A simplified analogy can be found in animals where diseases vs. fitness and prolonged lifespan vs. faster aging, are dependent on mitochondrial ROS production and ES, and body temperature is regulated by sweating, temperature-dependent respiration, and gene regulation. In this review, we discuss the universal features of stress factors, ES, the cellular production of ROS molecules, ROS scavengers, hormones, and other regulators that coordinate life and death.

## 1. Introduction

Commonly, organisms grow in non-optimal (stress) conditions. Oxidative stress appeared during the evolution of aerobic life as a consequence of the properties of oxygen and the enhanced accumulation of various reactive oxygen species (ROS). Organisms are capable of experiencing oxidative stress and surviving by overcoming environmental pressure. The altered redox state of cells triggers the activation of multiple defence and acclimation mechanisms; some of them allow for scavenging ROS directly and others induce the de novo synthesis of broken molecules in the cells or use ROS to induce signalling pathways. However, an organism that has to acclimate or adapt to external factors will have to prioritise survival rather than growth at the level of the cell, tissue, whole organism, or even the entire community. 

For plants, the inevitable consequences of oxidative stress, such as impaired crop quality and quantity, can occur. Understanding stress and its outcomes allows for the optimal cultivation of plants. This review provides a general overview of stress with particular attention paid to oxidative stress. Different types of plant stresses in the context of environment and organisms are listed. Despite the differences between the several definitions of (oxidative) stress, its universal character is considered comparing plant, animal, and bacterial stress responses. Among others, common ROS, their antioxidative antagonists, and their universal effect on the organisms are characterised. Considering their defined concentrations, it is shown that H_2_O_2_, O_2_^•−^, ^•^OH, and ^1^O_2_ determine various responses, finally lead to the induction, reduction, or inhibition of growth, and alternatively induce tolerance, acclimation, or defence against stress. In both cases, ROS excess can cause cell and organism death. Therefore, mechanisms of both acclimation and programmed cell death (PCD) are distinguished in the review. Unlike animals, plants cannot respond to the changes in their environment by moving to a more suitable environment, a feature that helps in surviving seasonal weather and resource changes. Therefore, plants also need to tolerate oxidative stress in a more specific manner in order to minimise its impact. A combination of a wide spectrum of molecular processes and complex regulatory mechanisms, including changes in gene expression and regulatory networks are described. In particular, molecular and physiological mechanisms of acclimation, which allow plants to survive future stress, and the importance of such mechanisms for the cell, tissue, whole organism, or even the entire community are underlined.

## 2. Stress

There is no general definition of stress in life sciences. In an environmental context, the term ‘stress’ refers to external factors disturbing the internal homeostasis of the organism. In the context of the organism, stress is considered as short- or long-term physiological, metabolic, and molecular alterations provoked by suboptimal internal or environmental factors [1,2]. Stress can be described by several attributes.

In an environmental context, stress is **(1)** heterogeneous; a large variety of factors can induce a stress status. There are abiotic factors—the physicochemical properties of the environment, and biotic factors—the interactions with the organisms [3]; factors of natural or anthropogenic origins. An example of a natural abiotic factor is atmospheric or soil conditions as a derivative of the climate (e.g., seasonal changes), while the biotic factor is represented by the qualitative and quantitative pressure between species in a natural environment (e.g., new pathogens and invasive species). Human activity results in abiotic stress, either directly (e.g., pollution, urbanisation) or indirectly (e.g., global warming, changes to the ecosystem). There are common biotic stresses (e.g., modification of the ecosystems by agricultures) and specific (e.g., dedicated GMOs or accidental release of pathogens from laboratories). Abiotic factors with anthropogenic origins can also influence natural biotic factors, and vice versa. For example, the major causes of poor quality of water and soil are an excess of phosphorus, nitrogen, and pesticides, which are consequence of intensified agricultural practices. Their presence facilitates invasive species, such as algae *Prymnesium parvum,* which kill fishes (e.g., in the River Odra, Poland, 2022). We can distinguish factors that cause short-term stress (factors with low intensity or rare), and chronic stress (factors with high intensity or in excess) [4]. Stress is **(2)** bipolar. The optimal factor level is a narrow range, and each deviation from the optimum in the direction to a lower or higher factor level induces stress in the organism. The same abiotic factor can have two extremes, e.g., light—low level or excess; temperature—cold or heat; water—drought or flooding; minerals—low nutrients content or salinity and high level of heavy metals; gaseous atmosphere—low or high level of CO_2_, O_2_, normoxia, hypoxia, etc. The biotic action of bacteria, viruses, fungi, or insects can be a competition, parasitism, or pathogenesis, but also they can also have a supporting effect, such as the protocooperation of nitrogen-binding rhizobacteria and mycorrhiza, and a beneficial interaction with endophytic organisms [5].

In an organismal context, stress is **(3)** unavoidable and **(4)** natural. Unstressed status is rarely known a priori. Organisms experience stress, and respond to it, while stress tolerance, acclimation, or adaptation can determine their potential to grow, develop, reproduce, and survive under particular conditions. Strategies to survive during stress require a balanced distribution of the energy between the signalling pathways responsible for growth or acclimation/defence responses [6,7]. Stress is **(5)** complex. The state of organisms results from a number of signalling pathways, because many different stress factors affect the organisms at the same time or the same factor occurring at different times has a long-lasting effect. Some signalling pathways overlap, as the energy required for separate mechanisms under each individual factor would be disproportional to the outcome of the defence or acclimation [6,7]. Stress is **(6)** multilevel. It has an impact at each level of the organism’s complexity: from molecular and physiological, through inter-organellar, intercellular, or inter-organs, to interactions between organisms (in the community) [8,9]. Considering the interaction between environmental factors and organisms, stress is **(7)** synchronous. The environmental factors fluctuate in their intensity, frequency, severity in time, and intervals, they affect organisms directly or indirectly, in a primary or secondary way, and they cause reversible (acclimation) or irreversible (adaptation or death) changes. Multiple signals can have additive effects, but they can also induce responses to a lower extent than each individual factor. Therefore, organism fitness is determined by synchronizing the metabolism and providing the most optimal responses [10,11]. Stress is **(8)** relative, as the same amount of stress can initiate a response depending on the organism’s status; in some cases, the same factor can have a negative, neutral, or beneficial effect. Low or moderate stress improves organism growth, development, and defence responses [12,13,14]. It results from metabolic stress, defined by the accumulation of metabolites, which in turn, influence the changes in hormones, reactive oxygen species (ROS), and gene expression profile [13]. For instance, ROS accumulation in the plant chloroplast and mitochondria induces retrograde signalling, and then systemic acquired acclimation (SAA) [13,14], defence through direct ROS effect on pathogens or systemic-acquired resistance (SAR) [13,15], and even induction of intra-species and inter-species network acquired acclimation (NAA) [9]. Periodic ROS increase and decrease (stress and optimal conditions) can promote more efficient stress metabolite accumulation, while the antioxidant application can impair some SAA, SAR, and NAA responses. The nature of these mechanisms in plants is similar to human and animal body fitness after exercise and muscle growth or immunity after a vaccination [16]. In contrast, excessive stress, exercise, or pathogen virulence results in the amount of ROS being inadequately neutralised by antioxidants. Then, ROS can cause cumulative damage to cellular proteins, lipids and nucleic acids, increase cell sensitivity, and lead to the ultimate end, i.e., cell death (CD). Stress is **(9)** a dynamic state, as stress factors impact the anti-stress processes inside the organism [17]. The type of cell mechanism (e.g., leading to adaptation, acclimatization, intervention, active defence) results from feedback between the external stress (type and level) and the actual internal state (capacity) of the organism, which in turn is an outcome of the growth conditions prior to the stress factor [7,9,12,13]. If the threshold of stress exceeds the ability of an organism to balance the metabolism, death occurs at the level of the cell, tissue, whole organism, or even the entire community.

Considering the methodological approach used to distinguish stress status, **(10)** stress is a method-dependent variable. Stress in living organisms, recognised at the level of chemical reactions, does not differ significantly from the reactions taking place in a test tube [18]; thus, stress can be analysed in a simplified and artificial system. A different methodological approach is applied, taking into account that an organism functions at four dimensions of complexity (3D structure and time). The multicellular (3D structure) organism controls active acclimation and defence (in time). As a result, the ‘fight or flight’ response can be initiated for the organisms that are able to move (e.g., animals, humans), or ‘defence-no-death’ response in organisms that are unable to move (e.g., plants). Further, in a more complex organism (e.g., animals), the ‘stress’ is used to describe the experience, which requires choosing a response strategy based on perception, calculation, and assessing the possibilities. In these cases, methods to study stress are extremely different from those applied at the lower level of organism complexity.

## 3. Oxidative Stress

Oxygen is a reactive chemical element that gradually accumulated in the atmosphere and in all organic molecules along with early life formation (between 2.8 and 1.8 billion years ago [19]). As a consequence, oxygen is present in the chemical reactions of the organism even at a low level of gaseous oxygen. Ground state oxygen is a diradical, and two unpaired oxygens have a spin state of 1/2 for a total resultant spin S of 1 and makes ground state oxygen a triplet. ^3^O_2_ remains inert towards organic compounds in its singlet state, owing to the resonance stabilisation of its π-electron system and Wigner’s spin conservation rule. This permits life to exist under aerobic conditions. However, the thermal, photochemical, or chemical activation of some biomolecules often leads to their excited triplet states, pro-oxidant activity, and release of ^1^O_2_ (and other free radicals). Their reactivity is greatly enhanced by the excess energy and the spin-allowed character of the reactions with molecules that display singlet multiplicity [20]. The redox (reduction/oxidation) imbalance induces oxidative damage to cell compounds and organisms. Therefore, cells have evolved control systems to cope with the oxidising factors. If homeostasis between oxidising agents and the agents inhibiting the accumulation of the oxidising agents is impaired, it can generate a state named oxidative stress. Organisms (e.g., mutants) that are unable to detoxify ROS have difficulty growing in the presence of oxygen [21].

Life on Earth shifted from anoxia to oxygen conditions [22], evolving the respiratory systems. The increasing ROS level allowed for more complex organism development, improved body size, and biodiversity. On the other hand, the system of the transfer of electrons to oxygen, which is the final electron acceptor in the electron transport reaction, is highly conserved among aerobic organisms, with ROS and antioxidants commonly produced even in obligatory anaerobes [23]. With this in mind and considering that all attributes of stress **(1–10)**, are caused by the oxidative stress in organisms of different biological kingdoms, one should expect that oxidative stress, to some extent, **(11)** is universal.

## 4. Reactive Oxygen Species (ROS)

Oxidative stress is induced by the limited pool of oxidising molecules. The most prominent ROS are hydrogen peroxide (H_2_O_2_), a non-radical species, and superoxide anion (O_2_^•−^), a highly reactive free radical. Additionally, hydroxyl radical (HO^•^) and singlet oxygen ^1^O_2_, a non-free-radical species, are involved in the oxidative reactions.

### 4.1. H_2_O_2_

A ‘non-radical“’H_2_O_2_ (without unpaired electrons) is produced in all aerobes studied to date, from prokaryotes to humans, provided water, organics, and light, are present. H_2_O_2_ is maintained under tight control at nano- or micro-molar levels (10^–5^–10^–9^ M); however, higher levels are found in the plants (10^–5^–10^–4^ M) [9,13], when compared to animals and humans (10^–10^–10^–9^ M) [24]. In cells, H_2_O_2_ has a half-life of ∼1–100 ms, and can diffuse over a distance of 1 μm (a standard distance between organelles, which function as relay stations) [24]. At physiological concentrations, H_2_O_2_ is transported through the tonoplast and chloroplast inner envelope (plants), and between cells through aquaporins that are present in the plasma membrane (in most of the species) [25]. ‘Physiological’ levels of H_2_O_2_ are produced, e.g., by cryptochrome, a blue light photoreceptor, which occurs in, e.g., *Arabidopsis* and *Drosophila* [25]. H_2_O_2_-mediated signalling is based on its increase to ∼10^–4^ in plants and ∼10^–6^–∼10^–7^ M in animals and humans. At these levels, H_2_O_2_ induces reversible oxidation, particularly of cysteine residues in proteins, thus leading to alterations in their allosteric structure or enzymatic function. A concentration of H_2_O_2_ exceeding the physiological levels (∼10^–5^ M) causes non-specific oxidation and damage to various molecules [25]. H_2_O_2_ easily oxidises reduced iron, and hence, damages the iron–sulphur clusters of enzymes, inactivates proteins using mononuclear Fe(II) as a catalytic cofactor, makes it difficult to provide iron into new metalloenzymes, and thus also disrupts iron metabolism. H_2_O_2_ also reacts with unincorporated Fe, generating HO^•^ radical, harmful to biomolecules, including DNA and lipid membranes. Chronic oxidative DNA damage leads to mutation, aging, or to carcinogenesis in mammals [26]. H_2_O_2_ accumulation at levels higher than 10 ^–5^ M inactivates enzymes of the Calvin cycle, such as fructose-1,6-bisphosphatase, sedoheptulose-1,7-bisphosphatase, and phosphoribulokinase, as well as most of the kinases and transcription factors containing thiolate residues. In *Arabidopsis,* 10^–3^ M H_2_O_2_ induces cell death and inhibits growth, while in mammals and yeast, toxicity occurs already at micromolar concentrations [25]. The main sources of H_2_O_2_ in plants and animals include (i) the flavin-dependent oxidases and xanthine oxidases in the endoplasmic reticulum, peroxisomes, and cytosol; (ii) the acyl-coenzyme A (acyl-CoA) oxidases in peroxisomes (fatty acid oxidation); (iii) superoxide dismutase in the mitochondria, nucleus, peroxisomes, and the extracellular matrix; and (iv) membrane-associated NADPH oxidases (NOXs in mammalian) and respiratory burst oxidase homologues (RBOHs in plants) that are located in various subcellular compartments [25,27,28,29,30]. In plants, additionally, extracellular heme-containing Class III peroxidases are a source of H_2_O_2_; however, some peroxidases can be inhibited by H_2_O_2_ by a negative feedback mechanism [28]. In chloroplasts, electron transport activity and superoxide anion dismutation produce H_2_O_2_ under stress. Several superoxide dismutase (SOD1–SOD3) also form H_2_O_2_ during plant photorespiration [29]. Glycolate, whose biosynthesis occurs in the chloroplasts, can be oxidised in leaf peroxisomes by glycolate oxidase (GOX) to glyoxylate and H_2_O_2_, mediating the communication between chloroplasts and peroxisomes through various metabolites [30]. H_2_O_2_-forming oxidases are also involved in polyamine and purine catabolism during the synthesis of hormones in plant peroxisomes and glyoxysomes [25]. In most cases, H_2_O_2_ is the result of the presence of other ROS, or results in the generation of other ROS [31,32,33].

### 4.2. O_2_^• −^

The intracellular concentrations of the O_2_^•−^ radical, produced by the one-electron reduction of molecular oxygen, are much lower (∼10^–11^–10^–12^ M) than that of H_2_O_2_ under optimal conditions. O_2_^•−^ has a half-life of less than 1 ms and can diffuse for a few micrometers from the site of generation [28]. It is difficult to distinguish the cellular effects of O_2_^•−^ from that of other ROS. The production of O_2_^•−^ increases the H_2_O_2_ level, as two O_2_^• −^ can react with each other and two H^+^ molecules to form H_2_O_2_. At the same time, an excited triplet chlorophyll can interact with O_2_ to generate singlet oxygen [6,25]. Although O_2_^• −^ is not a strong oxidiser, it readily reacts with the Fe-S structures of protein causing their malfunction and Fe^2+^ release, and then Fe^2+^ reacts with H_2_O_2_ to form the highly toxic HO^•^ radical (the Fenton reaction) [28,34]. The main source of O_2_^• −^ is the ‘leak’ of the mitochondrial electron transport chain [32,33,35,36]. Generation of succinate-dependent O_2_^• −^ and its dismutation to H_2_O_2_ was reported to be faster than pyruvate/malate-dependent ROS production, indicating a larger role for mitochondrial Complex II compared to Complex I [32]. The ubiquinone pool might serve as another site of ROS production in plant mitochondria. However, mitochondria are a major source of O_2_^• −^ in roots. Sites of ‘electron leakage’ in leaves are found in photosystem I and photosystem II [28,33]. At the electron acceptor side of photosystem II, pheophytin, primary quinone electron acceptor (QA), plastosemiquinone (PQH^•^), and cytochrome Cyt *b*559 are able to reduce O_2_ and form O_2_^•−^ [31]. At the electron-accepting (stromal) side in photosystem I, O_2_^• −^ is probably synthesised by the 4Fe–4S complex (clusters X) on psaA and psaB or A/B on psaC (Mehler reaction) [29,30,32]. O_2_^•^ can also be generated by xanthine oxidase in peroxisomes and cytosol, by nitric oxide synthase in Golgi apparatus, plasma membrane, and peroxisomes, by cytochrome P450 in the endoplasmic reticulum, by NADH/NADPH-dependent ETC in the plant peroxisomal membrane, and by NOXs and the flavin adenine dinucleotide (FAD)- or flavin mononucleotide (FMN)-dependent oxidases in mammalian peroxisomes [32,33,35,36].

### 4.3. ^•^OH

It is estimated that ^•^OH is commonly present at levels as low as ∼10^–15^—10^–16^ M, while the physiological threshold level is ∼10^–13^ M (the authors’ calculation on the basis of data from [37,38,39,40]). Due to lifetimes of ^•^OH up to ∼10^−9^–10^−6^ s and a short diffusion distance (~10^−9^ m), the radical acts at the place of its production [40] and thus cannot diffuse outside the cell and take a part in cell-to-cell signalling. ^•^OH can catalyse the scission of polysaccharides, while some organics react with ^•^OH by the abstraction of a proton to produce organic radicals (R^⁎^); they are highly reactive and further oxidised [40,41,42,43]. ^•^OH reacts with the polyunsaturated fatty acids of cell membranes and initiates the primary stage of lipid hydroperoxidation; thus, it generates a fatty acid radical (^•^Lipid), and a fatty acid peroxyl radical (LOO^•^). The LOO^•^ oxidises polyunsaturated fatty acid molecules, initiates new chain reactions, and produces lipid hydroperoxides (LOOH), which break down into more radical species [42]. With aromatic compounds, ^•^OH reacts within a double bond to form a hydroxy-cyclohexadienyl radical, which in the presence of oxygen gives a peroxyl radical, while with water forms phenoxyl-type radicals [41]. ^•^OH also cleaves to nucleic acids; the addition reactions yield radicals of DNA bases, whereas the allyl radical of thymine and carbon-cantered sugar radicals are formed from the abstraction reactions [43]. Under the excess amount of H_2_O_2_ and in the presence of Fe(II)), the Fenton reaction forms ^•^OH in the mitochondria, cytosol, nucleus, and peroxisomes. The Haber–Weiss reaction, catalysed by Fe ions, generates ^•^OH from H_2_O_2_ and O_2_^•−^ in mitochondria and cytosol. Importantly, ascorbic acid is likely to serve as a pro-oxidant reductant for Fe in the Haber–Weiss cycle in plants because its concentration is very high (1–20 mM). Similarly, it can be completed by the glutathione in a high concentration (0.2–5 mM). Together they form the ascorbate–glutathione cycle. ^•^OH is also directly generated from H_2_O_2_ (HOOH → ^•^OH + ^•^OH) and hydroperoxides (ROOH → ^•^RH + ^•^OH) provide light exposition. In plants under stress, ^•^OH is additionally generated by both photosystems in plant chloroplasts. In PSI, the leakage of electrons results in superoxide and dismutation to H_2_O_2_, then the formation of H_2_O_2_–Fe complexes of ferredoxin (inner-sphere electron transfer) [44]. In PSII, three transition metal-binding sites are involved in HO^•^ production from H_2_O_2_: (1) H_2_O_2_ reacts with free transition metals in the stroma; (2) non-heme Fe is involved through inner-sphere electron transfer; (3) heme- Fe of cyt b559 forms Fe–H_2_O_2_ complexes [28,44].

### 4.4. ^1^O_2_

^1^O_2_ represents the first excited electronic state (it is formed when the spin of the valence electrons of ^3^O_2_ is inverted) of molecular oxygen. ^1^O_2_ is not a free radical and does not carry a high-energy electron. In neutral conditions, ^1^O_2_ content is estimated at ∼10^−13^ M, its level causing cell membrane damage is ∼10^−8^ M, while a local concentration of ∼10^−5^–10^−4^ M leads to cell death (the authors’ calculation on the basis of data from [37,45,46,47]). Due to the short lifetime of ^1^O_2_ (∼4 us) in live cells, ^1^O_2_ can diffuse at a short distance of ∼10–250 nm from the place where it was created [48,49]. ^1^O_2_ causes rapid oxidative damages to pigments, proteins, lipids, and DNA [49,50,51]. ^1^O_2_, oxidizing C-C double bonds of aromatic amino acid residues in proteins, polyunsaturated fatty acids, guanine bases in nucleic acids, and thiol groups, forms hydroperoxides or endoperoxides [50]. These hydroperoxides initiate free radical chain reactions, e.g., upon light or interaction with hydroxyl radicals [51]. ^1^O_2_ can also interact with DNA by inducing breaks in DNA strands, causing chromosome defects and point mutations. Rapid lipid peroxidation, mainly linolenic acid, can directly precede cell membrane damage [50,51,52]. In plants, ^1^O_2_ also leads to photoinhibition due to the repair of the D1 subunit of PSII being inhibited. Altogether, cumulative damages to cell structures are considered as the main trigger of ^1^O_2_-induced cell death. On the other hand, although ROS can cause severe damage in living cells, increasing amounts of data have suggested that ROS-related cell death occurs mostly via the activation of signalling pathways leading to CD, while accidental CD due to oxidative injuries is a relatively rare phenomenon [49]. In animals, ^1^O_2_ sources include: (1) photooxygenation in mitochondria and cytosol; (2) myeloperoxidase in the cytosol; (3) lipoxygenase, dioxygenase, lactoperoxidase, and Fenton reaction in peroxisomes; (4) cytochrome P450 in endoplasmatic reticulum; (5) cytochrome C in mitochondria [53]. In plants in the light, most ^1^O_2_ is produced in the mesophyll cells of leaves from different sources: (1) the interaction of triplet oxygen ^3^O_2_ with a chlorophyll molecule in the triplet state at PSII reaction centre, when the electron flux is hampered [6,7,17,29,30,32,33,49]; (2) chlorophyll precursors (chlorophyllide and protochlorophyllide) and catabolites that function as photosensitisers during the hypersensitivity response [31,46,49]; (3) the cytochrome–b6f complex, the Fe-S cluster of Rieske proteins, as well as a chlorophyll with unknown functions [30,32,49,53,54]; (4) enzymatic reactions of heme proteins and lipoxygenases in cell compartments other than chloroplasts [42,44,48,49,53]; (5) phytoalexins synthesised upon biotic stress [49]. In the dark, ^1^O_2_ can also be formed through: (6) hydroperoxides from linoleic acid, which form tetrahydroperoxides and decompose with the generation of ^1^O_2_ according to Russell’s mechanism [48,49,52]; (7) hydroperoxides from linoleic acid, formed in reactions with hydroxyperoxyl radicals or hydroxyl radicals [49] and both types of reactions (6 and 7) occur during osmotic stress in the rhizodermis of the root tip, and later in the root apical meristems, leading to the death of root meristem cells and lateral root formation [48,49,50,55]; (8) peroxidase reactions in cell walls in response to stress [6,17,53]. 

## 5. Responses to Oxidative Stress

In each organism, the pool of oxidative/antioxidative mechanisms and molecules is dependent on the type and intensity of the stress (Table 1) [53]. Specific oxidative stress responses can be triggered by the activation of receptors. They convey defined signals into the cell by activating specific signalling pathways that ultimately affect cytosolic machineries or nuclear transcriptional programs. However, some factors can induce pleiotropic effects by common physiological signalling agents [7] and unspecific signalling pathways.

**Figure 1:**
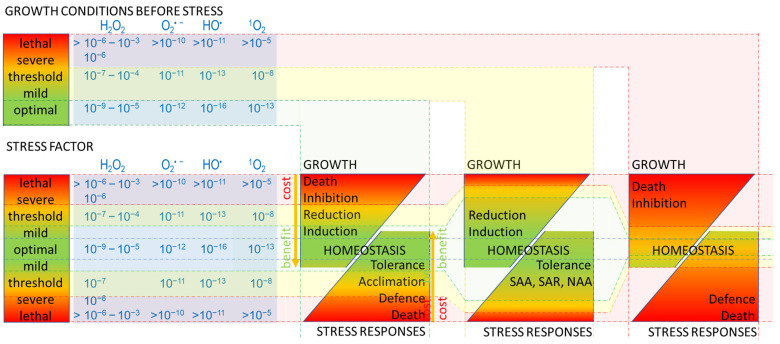
Response to the stress factor is determined by growth conditions prior to stress, the intensity of the stress factor, and ROS generated under stress. The higher intensity of the ‘stress’ factor ranging from optimal (green) to lethal (red), the higher is ROS level in cells and organisms (blue panel). ROS type (e.g., H_2_O_2_, O_2_^•−^, HO^•^, ^1^O_2_) and concentration (e.g., lower for animals and humans 10^−9^–10^−6^ M H_2_O_2_; than for plants 10^−6^–10^−3^ M H_2_O_2_) can influence the sensitivity of the organism, thus controlling its growth and stress responses from homeostasis (green) to death (red). ‘Homeostasis’ is a balance of metabolic processes regulating ‘growth’ and ‘stress responses’. ‘Mild stress’ can be a ‘benefit’ to cells and organisms; activates signalling pathways, and metabolic processes; and energy (yellow arrows) is used to ‘induce’ growth and maintain ‘tolerance’ response to stress [9,11,12,15,88,89]. Along with increasing the pressure of ‘stress’ up to the ‘threshold’ level, the redirection of metabolic energy, required for ‘acclimation’ and ‘defence’, results in lower energy availability for ‘growth’ (followed by its ‘reduction’ or ‘inhibition’); and vice versa, growth ‘induction’ limits ‘stress responses’. However, the effect of stress is still beneficial, as both ‘growth’ and ‘acclimation’ can be separate strategies to survive stress. Exceeding the ‘threshold’ stress impacts negatively; the ‘cost’ of either maintaining ‘growth’ or induction of ‘defence’ responses, or both, is too high for cells and organisms under ‘severe’ stress. ‘Inhibition’ of the growth has feedback through a further limitation of the metabolic energy supply. A ‘lethal’ level of stress leads to the ‘death’ of the cell and the whole organism. The ‘threshold’ of stress can be shifted, as it is dependent on the ‘growth condition before stress’. ‘Optimal’ growth conditions prior to stress factor ensure ‘benefits’ from a level of ROS, that are induced under no-stress (optimal) conditions or under ‘mild’ stress factor (green filling indicating beneficial growth and stress responses). ‘Mild’/‘threshold’ stress during ‘growth conditions before stress’ shifts the ‘benefit’ responses (wider green filling) and reduces ‘costs’ (thinner red filling), during the following stress event, and induces ROS-dependent systemic or network acquired acclimatization (‘SAA’, ‘NAA’) and systemic acquired defence (‘SAR’) [9,11,12,15,88,89]. In contrast, ‘severe’ or ‘lethal’ growth conditions prior to stress negatively impact growth and stress responses (growth inhibition and defence failure, leading to death) even at ROS levels induced under mild stress (wide red filling).

The unique responses are induced by specific factors, provided a stressor dominates or induces specific receptors. These individualised signals are dependent on the number, type, and combination of signalling molecules, the specific place, time, and period of molecule generation, the balance among activation, regulation, and termination of molecule biosynthesis, or a combination of all of these [87,88]. However, many of the ROS signalling pathways are interlinked and are commonly involved in the transduction and communication of redox signals in developmental processes (cell growth, differentiation, proliferation, senescence, aging, and apoptosis/CD) and physiological responses under a wide range of stress stimuli (including respiration and plant photosynthesis) [9,11,12,15,88,89]. ROS produced in different organelles (dependent on internal and external factors) may diffuse into the cytosol and trigger in a concentration-dependent manner common nuclear gene expression responses [90,91]. Particularly, the synthesis, stability, subcellular localisation, and/or activity of many specific transcription factors were shown to be regulated by different ROS and signalling pathways (in both epigenetic and transcriptional ways). Different ROS interactions may therefore determine broad-spectrum signalling that regulates growth/development and acclimation/defence (Figure 1). An outcome of changes in transcriptomic, proteomic, and metabolic networks leads to fitness and survival or to death.

In animals, a low level of H_2_O_2_ promotes cell proliferation and differentiation, while the mitochondrial accumulation of ROS serves as the central hub for signalling in inflammasomes, trained immunity, and immunometabolic pathways [53,92]. Low concentrations (1–10 μM) of H_2_O_2_ increase the viability and mitotic index of rat myoblasts and stimulate the proliferation of rabbit lens epithelial cells and primary human endothelial cells. H_2_O_2_ and other ROS accumulation in peroxisomes plays a vital role in cardiovascular and chronic kidney disease, hyperhomocysteinemia, metabolic syndrome, T cell-mediated inflammation, cigarette smoking stress, neurodegeneration, aging, and tumorigenesis [27,53,93]. At higher ROS concentrations, one of the most studied signalling pathways is the angiotensin II (AngII) activation of p38 MAP kinase. In the absence of AngII, the supraphysiologic level of H_2_O_2_ (100–200 μM) stimulates the phosphorylation of p38 MAP kinase, which in turn was shown to be inhibited by the application of extracellular catalase, or by overexpression of oligonucleotides anti-sense to the p22phox regulatory component of the NOX complexes. Many of the pro-growth effects of AngII are believed to be secondary to the activation of MAP kinases [25].

In plant cells, at low/moderate concentrations, ROS are secondary messengers in cellular signalling cascades that control several responses, e.g., gravitropism, seed germination, and lignin biosynthesis [94]. At higher concentrations, chloroplast-originated O_2_^• −^ and H_2_O_2_ take part in signalling networks that mediate biotic (pathogen attack, wounding) and abiotic (salinity, drought, hypothermia, hyperthermia, heavy metals, ozone, hypoxia) stress responses and lead to oxidative burst and programmed cell death [6,10,13,15,28,44,89]. H_2_O_2_ has been shown to induce the expression of genes related to most of such stresses through retrograde signalling pathways. Specifically, chloroplastic H_2_O_2_ can regulate genes controlling, e.g., stomatal formation (density), differentiation, and function (movement), thus influencing plant water use efficiency and biomass production [25,32,95]. The tight cooperation of H_2_O_2_ with MITOGEN-ACTIVATED PROTEIN KINASE 4 (MPK4, localised in the cytoplasm of guard cells) and abscisic acid (ABA) is crucial to the genetic control of the photosynthetic and thermal status of leaves, and the balance of the photosynthetic energy distribution either to growth or acclimation/defence responses [95]. In contrast, H_2_O_2_ originated from peroxisomes regulates genes involved in protein repair responses rather than direct photosynthesis processes [96]. O_2_^• −^ generation is observed under high light, UV, xenobiotics, and herbicide application. It directly modifies mitochondria or peroxisome ETCs, decreases the antioxidant pool, and increases the activities of NADPH oxidases and extra- and intracellular peroxidases. An increase in AtRBOHD and AtRBOHF expression is also required for the oxidative burst induced by pathogenic *Pseudomonas syringae* or *Hyaloperonospora parasitica* [97]. The ROS wave cell-to-cell signalling requires both apoplastic (RBOHs, and hydrogen-peroxide-induced calcium increase 1; HPCA1) and symplastic (Plasmodesmata-Localised Proteins 1 and 5; PDLP1 and PDLP5) functions. ROS can propagate between cells either through the apoplast or plasomodesmata; however, both compartments are needed for the cell-to-cell mobilisation responses [91,93,94,95,96,97,98,99]. The formation of ^1^O_2_ is favoured under limited CO_2_ availability due to the closure of stomata, during environmental stresses such as salinity, drought, and temperature extrema, as well as by the combination of these conditions with high-light stress [94].

On the other hand, the oxidative stress leading directly to CD is known in obligate anaerobic bacteria, growing in the absence of oxygen, such as *Clostridium*, *Bacteroides*, and the methane-producing archaea (methanogens) [98]. They are hypersensitive to oxygen, which poisons their key enzymes by O_2_^•−^/H_2_O_2_ produced in one-electron reduction of water and ^•^OH in the Fenton reaction. O_2_^•−^ and H_2_O_2_ are also generated by the autooxidation of bacteria flavoenzymes, NADH dehydrogenase II, lipoamide dehydrogenase, fumarate reductase, catechols, thiols, flavins, and oxidases under UV radiation. However, many studies have shown that anaerobic bacteria are not uniformly sensitive to oxygen, as incidental aeration is a common event in many habitats [100]. In facultative anaerobes, molecular oxygen impairs their metabolism in several ways: by direct quenching radical-based enzymes, oxidizing low-potential enzymic metal centres, and triggering rapid O_2_^•−^ and H_2_O_2_ formation [101]. There are known systems that mediate the cellular response to H_2_O_2_. In many Gram-negative bacteria (*Escherichia coli, Salmonella enterica, Caulobacter crescentus*) OxyR, a LysR family transcriptional factor, is the principal regulator for H_2_O_2_ response [101,102]. OxyR contains a regulatory domain, which senses H_2_O_2_, and a DNA binding domain, which modulates target gene expression directly. Similarly, PerR, an alternative transcription factor to OxyR, was found in Gram-positive bacteria (*Bacillus subtilis)* [103]. The regulon of PerR contains most of the same stress response genes as the OxyR regulon [104]. The same systems are important players in colonizing pathogens such as *Bacteroides fragilis* and *Hemophilus influenzae*. Mutants lacking *oxyR* were unable to colonise animals [101]. Further, H_2_O_2_ is generated and excreted by lactic acid bacteria to inhibit their competitors in proximity [105]. Bacteria also elicit ROS production, which damage the epithelial barrier. The introduction of a ROS scavenger significantly lowers oxidative damage, improves cell monolayer integrity, and reduces lipid peroxidation in mammalian cell-bacteria systems. Bacteria also have cytoplasmic systems to produce O_2_^•−^ against their competitors. A wide range of bacteria (and plants) secrete redox-cycling antibiotics, such as soluble quinones and phenazines. When transferred to target cells, they oxidise redox enzymes, and transfer the electrons to oxygen. On the other hand, enteric bacteria protect themselves from these compounds by activating the SoxRS regulon [101,106], elevating cytoplasmic SOD activity, pumping out the drugs, and modifying the membranes to reduce the entry [102]. Similarly, in the mammalian hosts, ROS production is induced as an antimicrobial defence; bacteria are impacted by the oxidative burst of phagocytes [106]. The role of NADPH oxidase in this process is important; humans and mice that lack it are susceptible to infections [101]. However, a few pathogens somehow tolerate the oxidative burst, e.g., the oxidative defence identified in *E. coli* is essential to the success of invading pathogens. The defensive system involving OxyR is highly effective and allows the growth of *E. coli* culture to survive in the presence of an extracellular H_2_O_2_ concentration that is 10^6^ times the normal intracellular H_2_O_2_ concentration produced by endogenous activity [104]. Similar to mammalians, plant hosts respond with superoxide anions generated by a dedicated NADPH oxidase to directly reduce microbial pathogen activity or indirectly confine the infection by strengthening the cell wall [10,11,107].

Altogether, ROS are produced in different organelles or cell compartments of different organisms (prokaryotic and eukaryotic) and can serve as retrograde signals involved in the regulation of the signalling metabolites to coordinate stress-response pathways as the crossroads of survival or death [108].

## 6. Antioxidative Systems—The Main Player in Oxidative Response Integration

**Figure 2:**
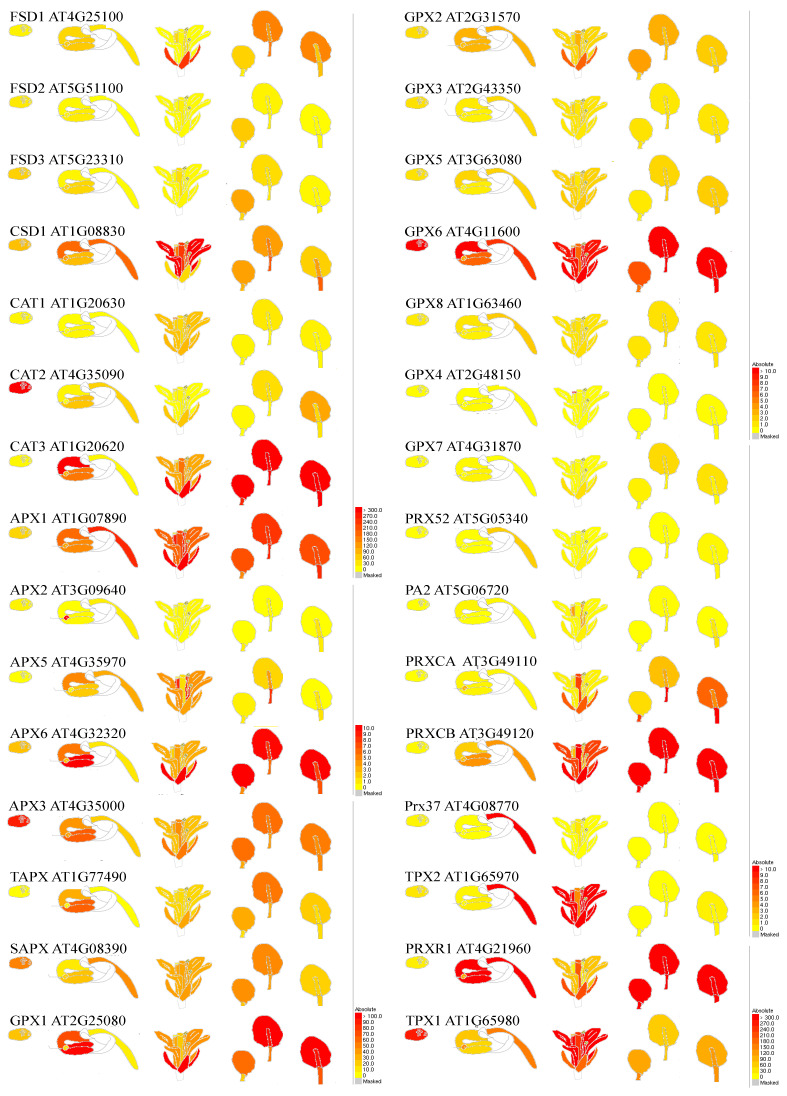
Developmental- and organ-dependent expression of genes coding superoxide dismutase, catalases, and peroxidases, based on Klepikova *Arabidopsis* Atlas eFP Browser at bar.utoronto.ca. Gene names and characteristics are provided in Table 2.

The steady-state levels of different ROS are largely determined by the efficiency of the antioxidative system consisting of specific components (Figure 2). They represent redox regulators and are involved in various processes of different cellular compartments [9,13,109]. However, the specific antioxidant responses are different from species to species, from organ to organ, and in some cases from cell to cell, for example, due to specific *cis*-regulatory element shuffling, but the presence of the general antioxidant defence is universal [110,111,112] (Figure 2). The antioxidants can delay or inhibit cellular damage in the intracellular and extracellular environment. A few ways to classify antioxidative molecules are at hand.

The most common is categorization according to the mechanism by which they are involved, i.e., enzymatic and non-enzymatic antioxidants, and correspondingly according to their size, i.e., large- and the small-molecule antioxidants. Enzymatic large-molecules antioxidants are enzymes that work by breaking down and removing individual radicals, or their cooperation can interrupt free radical chain reactions by converting oxidative products to H_2_O_2_ and then to H_2_O. The most known are superoxide dismutase (SOD, catalyses the removal of O_2_^• −^ by dismutation it into O_2_ and H_2_O_2_), catalase (CAT, converts the H_2_O_2_ into H_2_O and molecular oxygen), peroxidases (POX, works in the extra-cellular space for scavenging H_2_O_2_), glutathione peroxidase (GPX, catalyses the reduction of H_2_O_2_ and HO_2_ to H_2_O and lipid alcohols, using thioredoxin as an electron donor), glutathione reductase (GR, catalyses the reduction of oxidised dimeric glutathione GSSG to reduced monomeric glutathione GSH, glutathione S-transferases (GST), ascorbate peroxidase (APX, utilises ascorbate as specific electron donor to scavenge H_2_O_2_ to H_2_O), monodehydroascorbate reductase (MDHAR), and dehydroascorbate reductase (DHAR) (Figure 2). They are mainly involved in either preventing the Haber–Weiss reaction (H_2_O_2_ + O_2_^•−^ -> OH^-^ + O_2_ + ^•^OH), i.e., superoxide dismutase SOD (O_2_^•−^ + O_2_^•−^ -> H_2_O_2_ + O_2_) and CAT, POX, GPX (H_2_O_2_ + H_2_O_2_^-^ -> H_2_O + O_2_) or involved in the Foyer–Halliwell–Asada pathway (water-to-water cycle), i.e., chloroplast SOD, APX, DHAR, GR, GPX and others (which reduce the O_2_^•^ to H_2_O_2_ and further to H_2_O utilising the reducing potential of ascorbate, glutathione, and NADPH). They work in the presence of cofactors such as copper, zinc, manganese, and iron, which stabilise the transitional bond formation during the metabolising of intermediates [112,148,149]. These enzymes not only protect various components of the cells from damages, but also play an important role in plant growth and development by modulating cellular and sub-cellular processes such as mitosis and cell differentiation, senescence and cell death, detoxification of xenobiotics, regulation of enzymatic activities, synthesis of proteins and nucleotides, and expression of stress responsive genes.

Examples of the non-enzymatic antioxidants are ascorbic acid, α-tocopherol, carotenoids, glutathione, phenolic compounds, alkaloids, flavonoids, and free amino acids. The non-enzymatic antioxidants can be categorised as water-soluble present in the cellular fluids such as cytoplasmic matrix (e.g., ascorbate) and lipid-soluble predominantly located in cell membranes (e.g., α-tocopherol, carotenoids, lipoic acid). In plant cells, carotenoids, tocopherols, plastoquinols, and ascorbic acid are the main antioxidants.

### 6.1. Superoxide Dismutase

Enzyme superoxide dismutase (SOD, 1.15.1.1) plays a central role in the defence against oxidative stress in all aerobic organisms [149]. SOD belongs to the group of metalloenzymes and is present in most of the subcellular compartments that generate activated oxygen. Depending upon the metallic co-factors (Cu, Zn, Mn, Fe, and Ni) that are associated with SOD, it has different isoforms. Cu/Zn-SOD, Mn-SOD, and Fe- SOD have been reported in plants [112,150]. Cu/Zn-SOD is present in the cytoplasm, peroxisomes, chloroplast, and at extracellular locations (apoplast), Fe-SOD is present in the chloroplasts, and Mn-SOD is present in the matrix of the mitochondria and in peroxisomes [150]. The Cu/Zn-SOD in its native form is a homo-dimer (cytosolic) and homo-tetramer (chloroplast and apoplast); similarly, Mn-SOD can also exist as a homo-dimer or homo- tetramer in peroxisome and mitochondria. Ni-SOD has been reported in bacteria and cyanobacteria, but not in higher plants [112]. The Cu/Zn-SOD isoform is present in cytosol, chloroplast, peroxisome, mitochondria, and at an extracellular location (apoplast) and Fe-SOD in the chloroplast of the plants, whereas Mn-SOD is found in the matrix of the mitochondria and in peroxisomes (Table 2). Fe- and Mn-SOD evolved divergently while Cu/Zn-SOD evolved convergently. SODs are required to support aerobic life and it is suggested that they evolved together with oxygenic photosynthesis.

SOD activity has been reported to increase in plants exposed to various abiotic environmental stresses, including light, drought, and metal toxicity (Table 2). FSD2 and FSD3 scavenge ROS in the early chloroplast development stage and thus protect the chloroplast nucleoids from oxidation. The expression of SPL7 and its targets COPT2 and FSD1 was differently regulated in various light signalling mutants. Under copper deficiency, the expression of both targets decreased drastically in continuous darkness. Data have indicated that cadmium (Cd) elicits SPL7-dependent copper (Cu) deficiency responses by altering expression of COPT1, COPT2, COPT6, CSD1, CSD2, miRNA398 b/c precursors and FSD1. Enhanced SOD activity in response to the water deficiency was detected in various *Phaseolus vulgaris* cultivars, *O. sativa*, *Trifolium repens*, and saline stress in *Cicer arietinum*, *Solanum lycopersicum*, *C. arietinum*, and *A. thaliana*. MPK6, was also involved in the MKK5-mediated FSD signalling pathway in salt stress. Transgenic plants expressing miR398-resistant forms of CSD1, CSD2, and CCS under the control of their native promoters are more sensitive to heat stress. The mRNA levels of CSD1, but not CSD2, were negatively correlated with miR398 levels during ozone, salinity, and biotic stress. In vivo light-sheet microscopy resolves the localisation patterns of FSD1, a superoxide dismutase with functions in root development and osmoprotection. In the field condition, supplemental ultraviolet-B enhanced SOD activity in *Triticum aestivum* and *Munga radiata*, and caused various responses among *Glycine max* cultivars [112]. LSU1 interacts with the chloroplast FSD2 and stimulates its enzymatic activity in vivo and in vitro. *Pseudomonas syringae* virulence effectors interfere with this interaction and precludes re-localization of LSU1 to chloroplasts.

Similar scavenging systems are distributed through all biological kingdoms and in most cellular compartments, including the mitochondria, peroxisomes, and cytoplasm of eukaryote. The model bacterium *Escherichia coli* contains two SODs in its cytoplasm and one in its periplasm. Interestingly, some of these enzymes take advantage of the fact that iron can react with O_2_^•−^ and H_2_O_2_. The importance of these enzymes was revealed by genetic studies of *E. coli*. Mutants that lack cytoplasmic SODs or CATs were found to be unable to grow under oxygen conditions and show increased mutagenesis rates [101].

It is also known that mutations in genes encoding SOD that deregulate dimer formation or funneling of O_2_^• −^ to enzymatic reaction centre or mutations in SOD regulatory proteins such as NF-κB cause neurodegenerative disorders in animals and humans [151,152,153].

### 6.2. Catalases

Catalases are tetrameric hemoproteins catalyzing H_2_O_2_ decomposition to water and oxygen [154,155,156,157,158]. CATs are unique as they do not require a cellular-reducing equivalent [94,159,160]. According to their catalytic mechanisms, CAT enzymes can be categorised into two groups: monofunctional with dismutation activity and bifunctional with dismutation/peroxidation activities. Different genes have been assigned according to their structure. CAT1 genes are conserved in animals, plants, and bacteria. These CATs are primarily active in peroxisomes and glyoxysomes, the sites of high H_2_O_2_ generation and turnover [160]. In plants, CAT1 isoforms are present in the cytosol, chloroplast, and mitochondria [Table 2] of leaves and they participate in H_2_O_2_ scavenging during plant development, i.e., early seedling growth, photorespiration, and long-term heat tolerance. CAT1 has a role in the resistance to the hemibiotrophic bacterial pathogen *Pseudomonas syringae* via a constitutively activated salicylic acid pathway. Jasmonic acid promotes leaf senescence through the MYC2-mediated repression of CAT2 expression and plays an essential role in growth and day length-dependent oxidative signalling. CAT2 is found in vascular bundles and participates in lignification in response to ABA and senescence and general cell redox homeostasis during abiotic and biotic stresses (Table 2). SHORT-ROOT Deficiency alleviates the cell death phenotype of the *Arabidopsis catalase2* mutant under photorespiration-promoting conditions [160]. CAT3 is expressed in seeds and reproductive tissues and its activity is high during catabolism of fatty acids and glyoxylate cycle in glyoxysomes (Table 2). CAT3 expression is induced with age and corresponds to an accumulation of H_2_O_2_ in the vascular bundles. During plant infection with CMV, the host proteasome pathway is, at least partially, responsible for the degradation of CAT3 or CMV 2b that can interact directly with CAT3. During abiotic stress (e.g., drought) CAT3 activity is crucial [156,157,158] and CAT3 can mediate CPK8 functions in ABA-dependent stomatal regulation. Data suggest that the interaction of SOS2 with both NDPK2 and CAT2 and 3 reveals a point of cross talk between salt stress response and other signalling factors including H_2_O_2_. CAT3 was shown to be an LSD1 (lesion-simulating disease 1)-interacting protein. LSD1 interacts with all three CATs in vitro and in vivo, and this interaction requires the zinc fingers of LSD1. The CAT enzymatic activity was reduced in the *lsd1* (*Arabidopsis*) mutant, indicating that its activity was partially dependent on functional LSD1. Consistently, the *lsd1* mutant was more sensitive to the CAT inhibitor 3-amino-1,2,4-triazole than the wild type, suggesting that the interaction between LSD1 and CATs is involved in ROS generation in the peroxisome. Genetic studies revealed that LSD1 interacted with CAT genes to regulate excess light-dependent runaway CD and hypersensitive-type cell death. The accumulation of salicylic acid, ethylene, and ROS was required for CD regulation by the interaction between LSD1 and CATs [89,154,159,161]. The *cat1/2/3* triple mutants displayed severe redox disturbance and growth defects even under physiological conditions compared with wild-type and the *cat2/3* double mutants. CAT-deficient plants are susceptible to paraquat, salt, and ozone, but not during cold stress [155]. These results indicate that CAT activity deficiency cannot be complemented by other H_2_O_2_ scavenging enzymes and that CAT activity is somehow interconnected with specific retrograde and stress signalling pathways.

The imbalances in peroxisomal H_2_O_2_ metabolism have been associated with multiple oxidative stress-related human and animals disease states, and it can be linked to alterations in CAT activity [93,162,163,164,165,166,167,168]. CAT modulates the expression of numerous genes, i.e., CAT inhibition or overexpression can activate or inhibit the activity of NF-κB. Transgenic overexpression of CAT protects cells by an overall decrease in oxidative stress, a shift of the protein thiol/disulphide balance towards thiols, a decrease in nitric oxide synthase activity, lowering the nitration of key enzymes involved in energy metabolism, a decline in NF-κB signalling, and proapoptotic gene expression. It was shown to protect the heart from injury, dysfunction, and diseases, aging, and mortality, hypertension, albuminuria, tubulointerstitial fibrosis, and tubular apoptosis [93]. The overexpression of CAT may also dampen H_2_O_2_ signalling and sensitise human and animal cells to different stressors (e.g., hepatocytes and fibroblasts, alveolar macrophages) or reduce the growth of cells (e.g., rat aortic smooth muscle cells, human aortic endothelial cells, human MCF-7 breast cancer cells, A-375 amelanotic melanoma cells, human promyelocytic HL-60 cells) [93,162,163,164,165]. In contrast, CAT inhibition increases oxidative damage, enhances metalloproteinases production, and impairs mitochondria functions [93,163,164]. Cancer cells frequently produce elevated levels of ROS which act as pro-tumorigenic signals that promote abnormal cell growth, migration, resistance to apoptosis (CD), adaptations to hypoxia, and genetic instability. CAT inhibition has been associated with risk of many different cancers, while its overexpression (e.g., in MCF-7 mammary cancer cells) has been reported to result in a less aggressive phenotype cancer cells and an altered response to chemotherapy [93,165].

Considering that oxidative stress determines the fate of obligate anaerobic bacteria, it is also not surprising that anaerobes possess effective scavenging systems. Catalases (Kat) are found in OxyR regulons. In *Eschericha coli, Salmonella enterica,* and *Caulobacter crescentus*, OxyR positively regulates the expression of such *Kat* and *oxyR* null mutants are much more sensitive to H_2_O_2_ [169]. On the other hand, in *Corynebacterium diphtheria*, *Corynebacterium glutamicum R,* and *Shewanella oneidensis* the regulation is negative. As a result, the deletion of *oxyR* in the bacteria can lead to the activation of major Kat and enhance tolerance to H_2_O_2_ [170]. Mutants of *E. coli* that lack either *Kat* and peroxidase exhibit distinctive growth defects. Complementation of Kat activity in the mutant restored the ability of the mutant strain to survive in the presence of higher H_2_O_2_ levels showing that the KatB may play a role in oxidative stress tolerance in aerotolerant anaerobic bacteria.

### 6.3. Peroxidases

Plant peroxidases serve as the second line of the defence system that helps plants to cope with excess H_2_O_2_.Various mechanisms catalysed by POX can be distinguished, i.e., peroxidative, oxidative, and hydroxylic cycles [112,171]. Apart from its role in the catabolism of H_2_O_2_ and redox homeostasis, POXs play a diverse role in plant growth and development, e.g., they are involved in cell wall cross-linking (lignification, suberisation) and loosening, as well as auxin catabolism. The correlation between the stress memory and activity of POX (as well as SOD and CAT), and higher levels of the antioxidative enzymes, confirm the important role in long time acclimation and defence responses [9,10,13,14,90,99,112].

Glutathione peroxidase (GPX) catalyses the reduction of H_2_O_2_ and HO_2_ to H_2_O and lipid alcohols, respectively. In plants, this enzyme is a thiol-based (an organic compound containing the -SH group) enzyme and uses thioredoxin as an electron donor to palliate the damaging impact of H_2_O_2_ [112]. There are two main differences between plant and animal GPXs; first, plant GPX contains cysteine in the active site, while, in most of the metazoans, seleno-cysteine is present in the active site; second, thioredoxin is used in the regeneration of plant-oxidised GPX, and regeneration occurs via GSH in animals. The higher GPX activity was reported during various abiotic and biotic stresses in photooxidative and immune responses. GPX3 can scavenge ABA- or drought-induced H_2_O_2_, thus, act as a ROS sensor to transduce oxidative signals during ABA and drought stress signalling [134,172]. GPX1 and GPX7 have partially overlapping functions. The activity of these GPXs increased under the compatible interaction of *Plasmopara halstedii* and *Helianthus annuus,* whereas decreased under the incompatible interaction with a virulent strain. Similar findings have been observed during the rice-blast pathogen interaction [112].

Ascorbate peroxidase (APX) is class I heme-peroxidases and is known as ascorbate (AsA)-dependent peroxidase. This enzyme functions as a scavenger of H_2_O_2_ and sensor of redox alteration [129,173,174]. APX is regarded as one of the most widely distributed antioxidant enzymes in plant cells, located in cytosol, stroma, thylakoids, mitochondria, and peroxisomes (Table 2) [25,30,173]. APX as a key enzyme in the Foyer–Halliwell–Asada pathway, utilises ascorbate, a specific electron donor, to scavenge H_2_O_2_ to H_2_O with a concomitant generation of MDHA [173]. Cytosolic APX1 plays a key role in the acclimation of plants to a combination of drought and heat stress, tolerance to Se, Pb tolerance. APX1 from *A. graveolens* has the optimum temperature for its activity of 55 °C and the expression of *its* gene is significantly increased under drought stress. The APX1 mainly works through activating the expression of the ATP-bind cassette (ABC)-type transporters, at least partially through GSH-dependent PC synthesis pathway, and coordinated control of gene expression. Studies indicate that in *apx1/cat2* double-mutant, a DNA damage response is activated, suppressing growth via a WEE1 kinase-dependent cell-cycle checkpoint. APX1 is important for photoprotection during the early chloroplast development and mitochondria under light stress. Similarly, deficiency in APX2 results in a decreased tolerance to light stress, an enhanced tolerance to abiotic stresses (drought, salinity, chilling, metal toxicity, UV irradiation), stunted growth and enhanced sensitivity to oxidative stress. APX6 delays aging, senescence of leaves, desiccation, and germination of seeds. A role of APX6 in the regulation of the crosstalk between auxin, abscisic acid, and ROS [112,174]. Similarly, overexpression of the chloroplast tAPX gene increased tolerance to oxidative stress. In contrast, drought-susceptible wheat genotypes had higher APX and CAT activity, AsA content, and lower H_2_O_2_ and MDA content. In another study, the drought-tolerant maize genotype was tolerant to water stress remaining the lower H_2_O_2_ and MDA content together with increase in SOD, CAT, and POX activities [175].

Other POX are known to cooperate with SOD, CAT antioxidant enzymes, in part mediated by SA (Table 2). A significant increase in the activities of POX and CAT was observed in leaves infected with powdery mildew and mosaic virus, and in lines tolerant to biotic stress. PRX33/PRX34-generated ROS production is involved in pattern-triggered immunity in tissue culture cells. Besides their function in signalling pathways, different POXs are also involved in polymerization of suberin and lignin, important compounds of passive plant defence barriers in a cross-talk with signalling pathways of jasmonic acid. PRX33 and PRX34 are required for SA- and PAMP-triggered ROS production, which can take a part in defence against pathogens. In contrast to cytoplasm, which is a highly reduced and antioxidant-enriched alkaline compartment, the extracellular space is acidic and normally does not contain high levels of H_2_O_2_ scavenging CATs and POXs. This low activity of enzymatic ROS scavengers provokes H_2_O_2_ accumulation in the apoplast, promoting oxidative stress signalling [99]. POX works in the extracellular space for scavenging H_2_O_2_ (Table 2).

## 7. Cell Death Regulators

Cell death is a highly organised process and is the ultimate end of the cell in all unicellular and multicellular organisms. It is involved in the maintenance of cell homeostasis in various organs and tissues. CD can be classified according to the triggering stimulus, cellular context, and morphological criteria.

Bearing in mind the triggering stimulus (stress) exceeds a threshold (physiological) level, its impact on the cell is negative (Figure 1). The oxidative stress and ROS accumulation disturb cell homeostasis. The metabolic energy is dissipated as heat (plant NPQ, human and animal temperature rise), and thus is limited for other processes. Therefore, the cell uses the energy for ‘growth’ or ‘immunity’ (not both). A ‘lethal level’ of stress leads to the CD and death of the whole organism, as cells are unable to adjust the metabolism, i.e., ‘costs’ exceed cell potential to de novo synthesis of molecules for the repairing mechanisms. The threshold level of stress is dependent on the growth conditions preceding stress.

In the cellular context, CD types are divided into programmed (active) cell death (PCD, an autonomous and orderly process regulated by genes and the formation of signal amplification complexes in order to maintain the organism’s homeostasis) and accidental cell death (ACD, an uncontrolled process triggered by accidental injury stimuli).

Considering morphological criteria, the human and animal CD includes apoptosis, autophagy-dependent cell death, necroptosis, pyroptosis, ferroptosis, parthanatos, mitotic catastrophe, senescence, and others such as entosis, NETosis, lysosome-dependent cell death, alkaliptosis, oxeiptosis [176], while plant CD is mainly divided into vacuole-dependent cell death, necrosis, hypersensitive disease defence response, and PCD in starchy cereal endosperm and during self-incompatibility [177].

The earliest and most common ways of human and animal CD are apoptosis and autophagy, which maintain cellular homeostasis and regulate cell fate. They promote CD independently or by a complementary interaction. Apoptosis machinery requires sensors to monitor extracellular (extrinsic pathway) and intracellular (intrinsic pathway) stress factors as well as effectors, which are executioners of CD. During apoptosis the cell volume is reduced, the chromatin is condensed, the nucleus is segmented, the plasma membrane is blebbing, and the cell is fragmented into apoptotic bodies and finally degraded by lysosomal enzymes. Autophagy provides an important mechanism to survive short-term starvation as well as a mechanism for quality control. The mechanism triggers the degradation of non-essential cell components (removal of defective organelles) and products (transported back to the cytoplasm for their re-use in metabolism). Importantly, apoptosis and autophagy-dependent cell death are considered crucial subroutines of PCD, which could play a vital role in targeted therapy and regulation of cancer cell death [176]. Necroptosis, another common process, is CD mode driven by receptor-interacting serine/threonine kinase protein (RIPK) 1 and is characterised by an early increase in cytosolic Ca^2+^ concentration, ROS and RNS production, lipid degradation, activation of calpain family proteases, uncoupling of respiration, and a drop in ATP. Further, membrane perforation, loss of function of ion channels/pumps, high intracellular osmotic pressure, mitochondria and nucleus dysfunction, cell and organelle swelling, lysosomal release of active cathepsin proteases to the cytosol, and loss of intracellular content, occur. However, there is a lack of apoptotic or autophagic features. Pyroptosis associated with an inflammatory response is initiated by pathogen-associated molecular patterns (PAMPs) or sterile molecular patterns (DAMPs) and involves gasdermins proteins as the primary executor of CD. The activation of the pyroptosis pathway leads to the formation of holes in the cell membrane, and the release of cytoplasm. Ferroptosis is CD-triggered by an unbalance between Fe-dependent lipid peroxidation in mitochondria (ROS accumulation, Fenton reaction) and lipid repair by glutathione peroxidase 4 (GPX4). Although the cell membrane and nucleus are unbroken, mitochondria shrink following the increase in density of their membranes and decrease of their cristae. Poly (ADP-ribose) polymerase-1 (PARP-1)-dependent cell death (parthanatos) occurs in many pathological processes such as inflammatory injury. Abnormal activation of PARP-1 and overproduction of ADP ribose polymers (PAR) trigger the signal transduction through mitochondria to the nucleus and induce CD [176]. Mitotic catastrophe occurs if cells attempt to divide without proper repair of DNA damage. Cells can attempt several divisions, but DNA becomes unstable and does not support cell function. Senescence is a final loss of proliferation capacity related to aging and involves telomere shortening and DNA damage.

Just as human and animal cells involve different processes of CD, the way to CD in plants may also change. The plant does not display apoptosis. Stress often induces shrinkage of the plant protoplast, which is morphologically similar to apoptotic cell shrinkage. However, the cell wall prevents the disruption of the cells as well as the plasma membrane is damaged and does not form apoptotic bodies. Furthermore, plant proteases with caspase-like activity do not lead to apoptotic morphology [177]. On the other hand, animal cys-protease (responsible for triggering PCD) is similar to proteases in plants; particularly to the vacuole processing enzymes (VPEs) and papain-like cysteine proteases (PLCPs, metacaspase), which play a key role in PCD. The vacuole-dependent CD is common during tissue and organ development; and is initiated, provided the formation of actin cables, nuclear envelope disassembly, cell content disruption by an autophagy-like process (engulfment of the cytoplasm by lytic vacuoles), and release of hydrolases from collapsed lytic vacuoles. Execution of CD is a slow process, but crucial during plant development (aerenchyma formation, leaf perforations in the lace plant, petal senescence, xylem differentiation, formation of embryo-suspensor, pollen). In contrast, early rupture of the plasma membrane and shrinkage of the protoplast occur under abiotic stress and lead to necrosis, therefore bringing the characteristics of necrotic CD closer to human and animal necroptosis. PCD is associated with the hypersensitive response induced by a range of abiotic stresses, successful recognition of a biotrophic pathogen, or the development of necrotrophic pathogens. In this case, PCD has similar necrotic characteristics, but the necrotic features can be also accompanied by the features of CD [89,95,124,126,177].

While the mechanism of CD has already been well described in animals, the exact molecular processes leading to plant CD are still unexplained in detail. The emergent question arises as to why and how CD is triggered by ROS in some groups of cells, but the others stay intact during SAA or SAR. Chloroplasts are important players in ROS-induced CD; they induce retrograde signalling from chloroplast to nucleus consisting of at least: NPQ and electrical potential changes, and signalling molecules, e.g., ROS, salicylic acid (SA), abscisic acid (ABA), jasmonic acid (JA), ethylene (ET); all of them are important for acclimation and defence [109,178,179,180]. The CD mutant *lsd1*, which lacks the functional LSD1 (AT4G20380) displays the runaway CD (RCD) with the inability to restrict the CD boundaries, once CD is triggered. The RCD in *lsd1* is evoked by excess light (EL, red light activating P680, but not P700), root hypoxia, impeded stomatal conductance, low temperature, drought, UV radiation, or bacterial infection [15,124,126,181,182]. Therefore, LSD1 was proposed as a negative CD regulator that integrates different signalling pathways in response to abiotic (SAA) and biotic (SAR) factors [109], at least involving O_2_^• −^ produced by the plasma-membrane-bound NADPH oxidase (RBOHT), photorespiratory burst of H_2_O_2_, ET, SA, and ABA [15,100,181,182]. RCD phenotype of *lsd1* can be reversed in non-permissive light conditions by improved atmospheric CO_2_ content or in *lsd1/cao* double mutant *(cao* has a mutation in the chloroplast Signal Recognition Particle 43, cpSRP43, and has reduced light absorption capacity in photosystem II light harvesting complex, higher energy quenching capacity, higher NPQ) [181]. Furthermore, the expression of *Oryza sativa LSD1* ortholog (*OsLSD1*) is light-induced and dark-suppressed. ET is required during RCD in the *lsd1,* since the ET precursor (1-aminocyclopropane-1-carboxylic acid, ACC) is elevated in the mutant, while the mutation in *EIN2*, which encodes an ET receptor, inhibits RCD [15]. LSD1 also regulates SA levels in *Arabidopsis thaliana* and *lsd1* requires elevated levels of SA during stomatal closure [181]. Altogether, initiation and propagation of RCD in *lsd1* are dependent on the amount of light energy absorbed in excess by the PSII (P680) light-harvesting complex, deregulation of stomatal conductance, photorespiration, and ROS/hormonal perturbations, while LSD1 is associated with the chloroplast retrograde signalling, positive regulation of antioxidant machinery, and prevention of the pro-CD pathway below certain oxidative stress level [15,181]. LSD1 cooperates with ENHANCED DISEASE SUSCEPTIBILITY 1 (EDS1, AT3G48090) and PHYTOALEXIN DEFICIENT 4 (PAD4, AT3G52430). Both proteins are components in gene-mediated and basal disease resistance, activation and amplification of SA signalling, mediation of antagonism between SA and JA/ET pathways during defence responses. They are essential for RCD, since in double mutants *eds1/lsd1* and *pad4/lsd1,* RCD is imposed regardless of biotic or abiotic stress applied to plants [15,181,182]. LSD1 and EDS1 cause opposite effects considering ROS ethylene and SA accumulation under different adverse condition [15,124,126,182].

LSD1 can also directly interact with bZIP10 TF and then inhibits its movement to the nucleus. Functional bZIP10 is essential for *lsd1*-specific RCD and both, R-gene mediated and basal defence responses. Furthermore, ten additional putative LSD1 interactors were reported. Among others, Zn-finger domains of LSD1 can bind to a cysteine-dependent protease—metacaspase 1 (MC1), which was suggested as a positive regulator of CD, while null mutation in MC1 suppresses CD in *lsd1* background [183,184]. On the other hand, LSD1 can interact with GILP, a negative regulator of pathogen-induced CD. LSD1 consisting of three Zn-finger domains may also act as a transcription factor (C2C2 class of Zn-finger motifs with the homology to GATA1-type TFs and conserved consensus sequence: CxxCRxxLMYxxGASxVxCxxC) and directly bind to DNA and/or proteins [183,184]. However, LSD1 was not confirmed as a transcriptional regulator acting by itself. *lsd1* mutant had different phenotype and gene expression profiles in the ambient laboratory and non-permissive field conditions. Mutants developed RCD and had over 2100 genes deregulated in stable laboratory conditions, but RCD did not progress when the expression of only 43 genes was changed in natural field long photoperiod, variable light, and presence of UV radiation. It indicated rather the LSD1 role in transcriptional regulation [124,126].

These results suggest that LSD1 may in fact act as a scaffold protein, bringing together other CD molecular regulators. LSD1 under control of EDS1 and PAD4 conditionally regulates photosynthesis, transpiration (water use efficiency), ROS/hormonal homeostasis, CD, seed yield, and thus determines plant fitness [115,117], or LSD1 acting as conditional-dependent hub regulator and interactor of TFs, can modulate diverse cellular processes via CD regulation and/or plant acclimation to different stresses.

## 8. Acclimation—N–Death: SAA, SAR, NAA

ROS communication can occur between cells of different organs as systemic signalling [13,14,99]. The signalling towards acclimation of the distal organs (termed ‘systemic acquired acclimation’, SAA), was identified to be dependent on H_2_O_2_ signalling between excess light-challenged and unchallenged plant organs [14,89]. The evidence for systemic signalling in response to local wounding, heat, cold, salt and pathogen attack has also been published [6,10,12,13,14,89]. Systemic signalling after a local stress allows the whole plant to adjust gene expression and regulate many of the systemic processes essential for achieving SAA to abiotic stress, and SAR to pathogen attack [6,10,12,13,14,178,179,180]. SAA and SAR depend on a mechanism in which the local apoplastic production of H_2_O_2_ by the respiratory burst oxidase homolog D and F (RBOHD and RBOHF) proteins can trigger the production of ROS by neighbouring cells inducing a systemic autopropagating signal termed the ROS wave [180]. ROS, cooperating with Ca^2+^, electric signals, and hydraulic signals can be transmitted within seconds and minutes from the tissue of origin (local) to distant tissues (systemic) through the plant vascular bundles using xylem parenchyma and phloem cells. The activation of systemic membrane potential, calcium, ROS, and hydraulic pressure signals, in response to stress, is dependent also on glutamate receptor-like proteins 3.3 and 3.6. Further, systemic ROS signals were shown to be regulated by cyclic nucleotide-gated calcium channel 2 (CGNC2), mechanosensitive small conductance-like (MSL) channels 2 and 3, plasma membrane intrinsic protein 2;1 (PIP2;1), and plasmodesmata (PD)-localised proteins (PDLP) 1 and 5, during systemic responses to HL stress [9,66]. Ca^2+^-activated NADPH oxidase works together with ROS-activated Ca^2+^-permeable cation channels to generate and amplify stress-induced Ca^2+^ and ROS signals [9]. An increase of cytosolic Ca^2+^ causes an increase in O_2_^• −^ production and vice versa, O_2_^• −^ activates Ca^2+^ influx through ROS-activated cation channels [185]. This signalling requires also photosynthesis optimization, antioxidants (e.g., SOD, APXs, CATs) balancing, and retrograde signalling from chloroplasts to the nucleus [141,179]. Gene expression anlysis during SAA revealed that in response to signalling of the stress to different tissues and organs (the systemic response), an activation of systemic membrane potential, calcium, ROS and hydraulic pressure signals is a main mechanism inducing SAR [185].

An intact structure of the phloem is required for the activation of SAR since it is the path for communication between the tissues infected by the pathogen and the uninfected distal tissues. Molecules such as pipecolic acid play essential roles in the translocation of long-distance signals via the phloem and the amplification of the immunity signal. SAR is characterised by the induction of a faster and more effective response against biotic stress as the plant cells are activated prior to the stress by pathogens. It was initially discovered in studies on the interplay of plants with microorganisms such as *Pseudomonas siringae* and was related to a phenomenon called ‘cellular memory’ [13].

Taking into account above, H_2_O_2_ is recognised as a universal indicator of the physiological status, which can monitor signalling acclimation and defence response of the one plant (SAA, SAR), and in the plant community (NAA). H_2_O_2_ influences plant yield, fitness, and the spatial occurrence of different species in the community [9,13,186,187,188,189,190,191,192,193]. However, it is important to mention, that, H_2_O_2_ levels are highly fluctuating. In each case, it is necessary to determine the relationship between H_2_O_2_ concentration and species-specific potential and stress-dependent conditions, and in the background of different interactions with other endogenous signalling molecules. H_2_O_2_ steady-state level differs depending on genotype, type of stress factor, the intensity of environmental abiotic and biotic stresses, growth conditions, monitoring method, etc., and H_2_O_2_ levels can range from 5 × 10^−6^ M to 45,000 × 10^−6^ M in plants [9,13,186,187,188,189,190,191,192,193] (Figure 1, Table 3). Earlier results indicated that the function of the important regulatory genes as *LSD1*, *EDS1*, and *PAD4* are modified in laboratory and field conditions, and in turn, they can change the level of endogenous H_2_O_2_ (Table 3). Since the environment greatly influences the overall plant metabolism and signalling, it is not a surprise that *lsd1* displayed different H_2_O_2_ and SA concentrations, maximum efficiency of PSII, and water use efficiency compared to other *Arabidopsis* genotypes. Furthermore, increased foliar concentrations of H_2_O_2_ were observed in all genotypes grown in the laboratory compared with the field (Table 3, [186]). The concentration of SA was also significantly correlated with H_2_O_2_, considering different genotypes and conditions (Table 3). However, a surprisingly similar seed yield (which is the ultimate result of coping with stress factors, SAA and SAR responses, and fitness) was found in the (optimal) laboratory and (multi-stress) natural environment [186]. Altogether, it proved that *lsd1* is more tolerant to combined stress factors in natural environments (e.g., drought, high-light, biotic stresses) than wild-type plants. LSD1, together with EDS1 and PAD4, are responsible for the control of H_2_O_2_ and SA in the cell, however, the signalling and regulatory gene’s impact on survival and reproduction are highly dependent on conditions. In this way, a significantly smaller number of *lsd1* transcripts was deregulated in the field compared to the transcripts level in *lsd1* grown in the laboratory. On the basis of the results summarised in Table 3, one should emphasise that the function of molecular regulators (including H_2_O_2_) should be studied not only under stable laboratory conditions, but also in the face of challenges posed by various natural conditions (the environment abounding in multiple stresses), and that it is necessary to confirm H_2_O_2_ levels with different methods.

Similarly, similar to the SAA response to light and wound requires H_2_O_2_, cooperating with transmission of electrical, NPQ, and calcium signals between local and systemic tissues of the same plant, recently discovered network-acquired acclimation (NAA) employs these signalling components to signal danger between individual plants, of the same or other species [9]. New type of plant-to-plant aboveground direct communication involving electrical signalling together with NPQ and ROS propagating changes was detected at the leaves. Wounding or high light stress applied to a single dandelion leaf induces an electrical signal transmitted on the leaves’ surface to connected neighbouring plants. The signal propagates, provided wet conditions on the leaves’ surface, which ensure a closed circuit. Signalling results in systemic photosynthetic (photochemical and nonphotochemical quenching), oxidative (ROS), and molecular (gene expression) changes in connected plants. Therefore, electrical signals can function as a communication link between stressed (transmitter) and unstressed (receiver) plants that are organised in a network (community) of plants. The electric signal can also induce autopropagation of ROS signalling in the receiver plant that did not experience stress. Similarly, a local application of high light stress can induce a systemic stomatal closure to the whole canopy. These systemic responses were also dependent on an RBOHD-mediated ‘ROS wave’. Considering a Darwinian point of view, NAA could be a side effect of the internal signalling networks of each plant, or an evolutionary advantage to the plant. Although ES and ROS carry only simple information, it is important to notice that they determine the range of responses (physiological, biochemical, molecular) to a given stress in receiver plants. Considering that plants have evolved the capability to ‘communicate a stress perception’ with each other in a rapid manner (aboveground plant-to-plant ES and ROS signalling), it could provide an obvious benefit to the receiver plant. In a similar way, the surveillance of defence signals from neighbouring plants can be considered. Plants cannot afford to stop metabolism, ROS, or electrical signals, as these are internally needed, and if the state of a neighbouring plant can be easily monitored, plants may have therefore evolved to take advantage of these signals to survey their environment. Therefore, plants now are included in the group of organisms capable of using ES and ROS actively as warning and communication signals between individuals and to induce acclimation responses as a part of stress memory [9].

An analogy can be found considering the animal and human body’s response to stress. General adaptive syndrome, according to this system, occurs in the alarm reaction, when the stressor is first occurring. The body begins to gather resources to deal with the stressor. The nervous systems are activated, and hormones (cortisol, adrenaline, norepinephrine) are release into the bloodstream to adjust body processes. These hormonal adjustments increase energy-levels, increase muscle tension, reduce sensitivity to pain, slow down the digestive system, and cause a rise in blood pressure. Finally, the stress affects working memory [194]. Ultimately, communication between individuals of the same species or of different species determines social responses, albeit at a much higher level of complexity.

## 9. Conclusions

ROS are the harmful by-products generated during normal cellular functions, but they are also important and universal signalling molecules in biological systems. Antioxidants contribute to maintaining ROS homeostasis and functioning under a stress (directly or indirectly leads to the overproduction of ROS). Tightly controlled ROS type and concentrations, together with ES, calcium ions, different hormones, and other cellular regulators, are functionally communicated between organelles, organs, and even organisms. Based on ROS-dependent retrograde signalling within one organism, various metabolic pathways can drive the cross-talk between different stress factors (in the fluctuating environment) and induce acclimation to subsequent abiotic stress, and resistance to biotic stress. Thus, ROS determines cell division or cell death, and organism life or organism death. Based on ROS-, and ES-dependent communication between organisms, ROS- and ES-induced signals in stressed organisms can determine the fate of the whole community. Therefore, ROS can work as a universal integrated network for sensing, alarming, and controlling stress.

However, the most critical aspects that need to be resolved are (i) the identification and functional dissection of redox-sensitive proteins that can be reversibly oxidised by ROS and serve as molecular ROS ‘receptors’; (ii) the mechanism applied to control of molecules involved in the transport of H_2_O_2_ across the membranes and from cell to cell, and its autopropagation; (iii) the biological consequences of changes in ROS metabolism on crosstalk in cellular signalling networks that drive physiological or pathological responses; (iv) how the ROS signal regulates two opposing processes, i.e., cell death and acclimatisation/adaptation.

Up to this time, the relation between ROS and electrical signal transmission between different organisms has been observed almost exclusively in aquatic or amphibious animals (e.g., sharks, rays, bony fish, and dolphins), because water is a much better conductor than air; however, now we can ask the question: have plants joined the group of organisms capable of using surface ROS and electric signals as a warning and communication signals between individuals?

## Figures and Tables

**Table 1:** Generation and localisation of ROS during biotic and abiotic stress in plants. Ap—apoplast; Ch—chloroplast; Ct—cytosol; Mt—mitochondrion; Px—peroxisome.

Plant Species	Stress Factor	Cell Compartment	References
**Hydrogen peroxide/Biotic stress**			
*Arabidopsis thaliana*	*Pseudomonas siringae*	Ap, Ch, Ct	[13,56,57]
*Glycine max*	*Pseudomonas syringae*	Ch, Ct	[58]
*Lycopersicon esculentum*	*Botrytis cinerea*	Mt	[59]
*Nicotiana tabacum*	*Pseudomonas syringae*	Ch, Ct	[60]
*Phaseolus vulgaris*	*Colletotrichum lindemuthianum*	Px, Ch	[61]
*Saccharum officinarum*	*Sporisorium scitamineum*	Px, Ct	[62]
*Triticum aestivum*	*Powdery mildew*	Ct	[63]
**Hydrogen peroxide/Abiotic stress**			
*Amaranthus tricolor*	drought, salinity	Px, Ch	[64]
*Arabidopsis thaliana*	high light, excess light, ammonium	Ap, Ch, Ct	[13,14,65,66]
*Cicer arietinum*	drought	Ct, Px	[67]
*Nicotiana tabacum*	freezing	Px, Ch	[68]
*Oryza sativa*	salinity, heavy metal	Px, Ch	[69]
*Saccharum officinarum*	hyper-osmotic	Ct	[70]
*Triticum aestivum*	drought stress	Ch	[71]
*Zea mays*	osmotic, drought	Ct, Px	[72]
**Superoxide anion/Biotic stress**			
*Glycine max*	*Pseudomonas syringae*	Ct	[73]
*Nicotiana tabacum*	*Mosaic virus*	Ct	[74]
**Superoxide anion/Abiotic stress**			
*Amaranthus tricolor*	drought, salinity stress	Ct	[75]
*Nicotiana tabacum*	high light	Ct	[76]
*Oryza sativa*	herbicide stress, heavy metal	Ct	[77]
*Triticum aestivum*	drought stress, high temperature	Ct	[78]
*Zea mays*	drought stress	Ct	[79]
**Hydroxyl radical/Abiotic stress**			
*Spinacia oleracea*	cold	Ct, Ch	[80]
*Vicia faba*	UV-B radiation	Ch	[81]
**Singlet oxygen/Abiotic stress**			
*Arabidopsis thaliana*	high light	Ct	[82,83,84,85]
*Nicotiana tabacum*	herbicide	Ct	[86]
*Pisum sativum;*	UV	Ch	[86]

**Table 2:** Gene Ontology (TAIR) for selected antioxidative enzymes (*A. thaliana*). Localisation: Ap, apoplast; Ch, chloroplast; Cm, cytoplasm; Ct, cytosol; Cw, cell wall; En, endosome; Er, endoplasmic reticulum; Ga, Golgi apparatus; Mt, mitochondrion; N, nucleus; Nd, nucleoid; Pd, plasmodesma; Pm, plasma membrane; Px, peroxisome; R, ribosome; Sm, stroma; St, stromule; Sv, secretory vesicle; Th, thylakoid; V, vacuole. Blast hits: Arc, Archae; Bac, Bacteria; Met, Metazoa; Fun, Fungi; Pla, Plants; Vir, Vir; Euk, other Eukaryotes.

Enzyme	Localisation	Biological Process, Response	Blast Hits	References
FSD1, AT4G25100 Fe superoxide dismutase 1	Mt, Ch, Pm	oxidative stress, light, O_3_, Cd, Cu, circadian rhythm	Arc 798; Bac 22429; Met 974; Fun 991; Pla 531; Vir 0; Euc 9610	[113]
FSD2, AT5G51100 Fe superoxide dismutase 2	Ch, Nd	UV	Arc 194; Bac 8106; Met 433; Fun 799; Pla 399; Vir 1; Euc 1590	[114]
FSD3, AT5G23310 Fe superoxide dismutase 3	Ch, Nd	removal O_2_^• −^	Arc 0; Bac 0; Met 736; Fun 347; Pla 385; Vir 0; Euc 339	[115]
CSD1, AT1G08830 Cu/Zn superoxide dismutase 1	Ct, Cm, N	UV, O_3_, Cu, Fe, light, salt, sucrose, bacterium, gene silencing by miRNA	Arc 6; Bac 2000; Met 1249; Fun 303; Pla 666; Vir 142; Euc 339	[116]
CAT1, AT1G20630 catalase 1	Mt, Ct, R, Cw, Px, Ch	multistress, photosynthesis, hypersensitive cell death, germination, stomata regulation, senescence, nutrients	Arc 22; Bac 4283; Met 677; Fun 546; Pla 461; Vir 0; Euc 119	[117]
CAT2, AT4G35090 catalase 2	Mt, Ct, R, St, Px	multistress, redox, N, P, S, photoperiod, heavy metals, cold, heat, light, senescence, cell death, pathogen	Arc 22; Bac 4292; Met 675; Fun 546; Pla 461; Vir 0; Euc 118	[118,119]
CAT3,AT1G20620 catalase 3	Ap, Ch, Cm, Ct, R, N, V, Cw, Mt, Px, Pm, Pd	N, P, S starvation, senescence, cold, drought, viruses, light	Arc 12; Bac 1396; Met 17338; Fun 3422; Pla 5037; Vir 0; Euc 2996	[120,121,122]
APX1, AT1G07890 ascorbate peroxidase 1	Ct, Cw, Ch, Pm	embryo, seed development, lignin biosynthesis, heat, heavy metals, multistress, photosynthesis, SAA, SAR	Arc 75; Bac 2912; Met 21; Fun 794; Pla 3291; Vir 0; Euc 1718	[123,124]
APX2, AT3G09640 ascorbate peroxidase 2	Ct, Ch	photosynthesis, SAA, SAR	Arc 55; Bac 2395; Met 20; Fun 832; Pla 3373; Vir 0; Euc 1764	[125,126]
APX3, AT4G35000 ascorbate peroxidase 3	Ct, Px, V, Mt, Ch, Pm, Pd	growth, development	Arc 86; Bac 3261; Met 20; Fun 794; Pla 3685; Vir 0; Euc 2017	[127,128]
APX5, AT4G35970 ascorbate peroxidase 5	Ch, Mt, Px, Pm	growth, development	Arc 103; Bac 4136; Met 9; Fun 795; Pla 3885; Vir 0; Euc 2440	[127,128]
APX6, AT4G32320 ascorbate peroxidase 6	Ch, Ct	seed germination, maturation	Arc 53; Bac 2233; Met 2; Fun 806; Pla 4037; Vir 0; Euc 933	[129,130]
TAPX, AT1G77490 thylakoidal ascorbate peroxidase	Ch, Th	retrograde signalling, cold acclimation, H_2_O_2_ signalling, response to ROS	Arc 55; Bac 2313; Met 5; Fun 620; Pla 3345; Vir 0; Euc 1471	[123,131]
SAPX, AT4G08390 stromal ascorbate peroxidase	Ch, Sm, Mt	response to oxidative stress, oxidation reduction	Arc 60; Bac 2389; Met 388; Fun 725; Pla 3386; Vir 0; Euc 2849	[132]
GPX1, AT2G25080 glutathione peroxidase 1	Ch, V	photooxidative tolerance and immune responses	Arc 2; Bac 4003; Met 799; Fun 210; Pla 390; Vir 8; Euc 2484	[133,134]
GPX2, AT2G31570 glutathione peroxidase 2	Cm, N, Px, Pm	salicylic acid binding	Arc 2; Bac 3597; Met 796; Fun 210; Pla 383; Vir 8; Euc 2467	[133]
GPX3, AT2G43350 glutathione peroxidase 3	Ga, Ch, Ct, Er, En, Mt		Arc 2; Bac 3505; Met 790; Fun 210; Pla 383; Vir 8; Euc 2426	[133,135]
GPX4, AT2G48150 glutathione peroxidase 4	Ct		Arc 2; Bac 3554; Met 785; Fun 210; Pla 383; Vir 8; Euc 2404	[133,135]
GPX5, AT3G63080 glutathione peroxidase 5	Ch, Ct, En, Er, Pm		Arc 2; Bac 3480; Met 788; Fun 210; Pla 381; Vir 8; Euc 2433	[133,135]
GPX6, AT4G11600 glutathione peroxidase 6	Ch, Ct, Mt, Pm	Pb	Arc 2; Bac 3728; Met 790; Fun 210; Pla 383; Vir 8; Euc 2480	[136]
GPX7, AT4G31870 glutathione peroxidase 7	Ch	immune responses	Arc 4; Bac 4124; Met 797; Fun 210; Pla 405; Vir 8; Euc 2493	[134]
GPX8, AT1G63460 glutathione peroxidase 8	Cm, N	DNA protection	Arc 2; Bac 3448; Met 795; Fun 210; Pla 387; Vir 8; Euc 2414	[137,138]
PRXCA, AT3G49110 peroxidase CA	Ct, Ap, Cw, V, Sv	pattern-triggered immunity	Arc 0; Bac 0; Met 3; Fun 31; Pla 4239; Vir 0; Euc 49	[57,139]
PRXCB, AT3G49120 peroxidase CB	Ga, Ap, Cw, Ct, V, Sv	pattern recognition receptor signalling pathway, light, unidimensional cell growth	Arc 0; Bac 0; Met 4; Fun 24; Pla 4218; Vir 0; Euc 36	[57,139]
Prx37, AT4G08770 peroxidase superfamily protein	Ap, V	growth, differentiation	Arc 0; Bac 0; Met 3; Fun 40; Pla 4262; Vir 0; Euc 43	[140,141]
PRX52, AT5G05340 peroxidase superfamily protein	Ga, Ap, Cw	xylem, lignification	Arc 0; Bac 0; Met 736; Fun 347; Pla 385; Vir 0; Euc 339	[142,143]
PRXR1, AT4G21960 peroxidase superfamily protein	Cw	lignification, defence	Arc 12; Bac 1396; Met 17338; Fun 3422; Pla 5037; Vir 0; Euc 2996	[143]
PA2, AT5G06720 peroxidase 2	Ga, Ap	cell elongation, defence	Arc 0; Bac 0; Met 736; Fun 347; Pla 385; Vir 0; Euc 339	[144]
TPX1, AT1G65980 thioredoxin-dependent peroxidase 1	Ch, Cm, Ct, N, Pm		Arc 11; Bac 1524; Met 175; Fun 308; Pla 230; Vir 0; Euc 1734	[145,146]
TPX2, AT1G65970 thioredoxin-dependent peroxidase 2	Cm		Arc 11; Bac 1524; Met 175; Fun 308; Pla 235; Vir 0; Euc 1742	[147]

**Table 3:** Endogenous hydrogen peroxide (H_2_O_2_) and salicylic acid (SA) levels in plant leaves of different species and genotypes in stable optimal (laboratory) conditions and under abiotic and biotic stresses.

Genotype	Conditions					References
	Optimal		Stress			
	SA	H_2_O_2_		SA	H_2_O_2_	
	(×10^−6^ M)			(× 0^−6^ M)		
*Arabidopsis Ws-0*	25	38.00	field	23.0	8	[186]
*Arabidopsis lsd1*	126	75.00	field	69.0	45	[186]
*Arabidopsis eds1*	36	37.00	field	25.0	7	[186]
*Arabidopsis pad4*	42	37.00	field	27.0	7	[186]
*Arabidopsis eds1/lsd1*	23	33.5	field	24.0	11	[186]
*Arabidopsis pad4/lsd1*	24	34.0	field	21.0	10	[186]
*Arabidopsis Ws-0*	14	4000	UV	16	5000	[187]
*Arabidopsis lsd1*	53	5500	UV	607	30,000	[187]
*Arabidopsis eds1*	19	2500	UV	54	6000	[187]
*Arabidopsis pad4*	46	3000	UV	64	12,000	[187]
*Arabidopsis eds1/lsd1*	25	2000	UV	28	4500	[187]
*Arabidopsis pad4/lsd1*	29	3800	UV	35	6500	[187]
*Arabidopsis Col-0*		~40	excess light		80	[13]
*Arabidopsis Col-0*	552	53	wounding	658	152	[9]
*Arabidopsis Col-0*	552	57	NAA/SAA	772	71	[9]
*Salvia miltiorrhiza*		200	SA		~6000	[188]
*Salvia miltiorrhiza*		200	H_2_O_2_		~5000	[188]
*Salvia miltiorrhiza*		200	SA + catalase		~1000	[188]
*Stylosanthes guianensis*		120	38°C		500	[189]
*Salix sp., Robinia sp., Ailanthus sp.*		25,000–45,000	drought		15,000–45,000	[190]
*Arabidopsis Col-0*		~8	*Pseudomonas syringae* pv. *tomato* DC3000		~30	[191]
*Phaseolus vulgaris*		~150	Pseudomonas *syringae* pv. *phaseolicola*		550	[192]
*Phaseolus vulgaris*		~150	*Botrytis cinerea*		300	[192]
*Lupinus luteus*	~5	~150	*Fusarium oxysporum* f. sp. *lupini*	14	400	[193]

## Data Availability

Not applicable.
